# Identification of blood-derived candidate gene markers and a new 7-gene diagnostic model for multiple sclerosis

**DOI:** 10.1186/s40659-021-00334-6

**Published:** 2021-04-01

**Authors:** Xin Chen, Huiqing Hou, Huimin Qiao, Haolong Fan, Tianyi Zhao, Mei Dong

**Affiliations:** Department of Neurology, The Second Hospital of Hebei Medical University, Shijiazhuang, 050000 Hebei China

**Keywords:** Biomarker, Support vector machine approach, Multiple sclerosis, Bioinformatics, Protein–protein interaction

## Abstract

**Background:**

Multiple sclerosis (MS) is a central nervous system disease with a high disability rate. Modern molecular biology techniques have identified a number of key genes and diagnostic markers to MS, but the etiology and pathogenesis of MS remain unknown.

**Results:**

In this study, the integration of three peripheral blood mononuclear cell (PBMC) microarray datasets and one peripheral blood T cells microarray dataset allowed comprehensive network and pathway analyses of the biological functions of MS-related genes. Differential expression analysis identified 78 significantly aberrantly expressed genes in MS, and further functional enrichment analysis showed that these genes were associated with innate immune response-activating signal transduction (p = 0.0017), neutrophil mediated immunity (p = 0.002), positive regulation of innate immune response (p = 0.004), IL-17 signaling pathway (p < 0.035) and other immune-related signaling pathways. In addition, a network of MS-specific protein–protein interactions (PPI) was constructed based on differential genes. Subsequent analysis of network topology properties identified the up-regulated CXCR4, ITGAM, ACTB, RHOA, RPS27A, UBA52, and RPL8 genes as the hub genes of the network, and they were also potential biomarkers of MS through Rap1 signaling pathway or leukocyte transendothelial migration. RT-qPCR results demonstrated that CXCR4 was obviously up-regulated, while ACTB, RHOA, and ITGAM were down-regulated in MS patient PBMC in comparison with normal samples. Finally, support vector machine was employed to establish a diagnostic model of MS with a high prediction performance in internal and external datasets (mean AUC = 0.97) and in different chip platform datasets (AUC = (0.93).

**Conclusion:**

This study provides new understanding for the etiology/pathogenesis of MS, facilitating an early identification and prediction of MS.

## Background

Multiple sclerosis (MS), which is a chronic inflammatory disease at the central nervous system of autoimmune etiology [1], is characterized by varying degrees of demyelination and axonal loss. MS predominantly affects young women (between the ages of 20 and 40) and is a leading cause of disability among young adults in the United States [2]. About 2.5 million cases have been reported all over the world, with about 400,000 taking place in the United States, moreover, the number of cases is expected to increase in the future [3–5]. Multiple factors including Epstein-Barr virus (EBV) infection [6], Vitamin D deficiency [7], smoking [8], and a high sodium diet [9] all contribute to the risk of developing MS. The pathogenesis of MS involves an immune attack against central nervous system (CNS) antigens, resulting in a sustained auto-reactive T-cell Peripheral activation [10, 11], Then post-activation myelin-reactive T Cells are able to penetrate the blood–brain barrier (BBB) into the central nervous system [12] to recruit other inflammatory cells, including T cells, monocytes, and B cells[13]. Long-term activation of microglia and macrophages will lead to destruction of myelin [14], and the activate resident glial cells such as microglia will cause persistent inflammation [15]. So far, however, immunomodulator therapy and symptomatic treatment are considered as two main strategies for treating MS, but they can only improve the body functions [16]. Several studies have shown that susceptibility to MS is genetically dependent [17, 18], but the specific genetic factors remain largely unknown. Therefore, the identification of risk alleles or candidate genes that play important role in the pathogenesis of MS remains a challenge.

MS, which is a chronic, progressive, immune-mediated disorder of the CNS, is characterized by neurodegeneration resulted from inflammation, demyelination and Axonal damage [19]. Currently, we still lack early diagnosis and management of MS [20], thus, biomarkers effective for MS identification are urgently needed. Bioinformatics analysis of gene expression profiles facilitate the screening of MS biomarkers. Dorothee Nickles et al. [21] compared the gene expression profiles of whole-blood RNA samples derived from both healthy individuals and MS patients, and identified abnormal individual transcripts and biological pathways in MS patients. Jeffrey M Trent et al. [22] performed gene expression profiling on peripheral blood mononuclear cells for identifying MS-related candidate genes by cDNA microarrays; Teresa Maria Creanza et al. [23] adopted differential network approach and demonstrated that MS networks showed a low connectivity relative to health status, and they also proved that interferon treatment can activate gene transcription. These studies have demonstrated the feasibility of screening key biomarkers for MS based on gene expression profiles. They designed different analysis strategies and got different results. It is well known that there are multiple optimal solutions for high-dimensional data analysis, but it is worth mentioning that these studies have focused on a single dataset of MS patients, in comparison, gene expression profiling of large samples from multiple cohorts to identify biomarkers will be more reliable in developing new biomarkers for early prevention and management of MS.

Biomarkers for MS can help diagnose, predict the disease course or determine the outcome of treatment response. Although biomarkers and extensive research are needed to identify them, the validation and clinical application of biomarkers in multiple sclerosis remains unmet. There is still a large gap between exploratory biomarkers proposed in many studies, proven biomarkers, and biomarkers incorporated into routine clinical practice.

This study used high-throughput gene expression profiles from a large cohort of MS patients to investigate the alterations of expression profiling patterns between MS patients and healthy individuals, aiming to identify potential biomarkers and to develop a diagnostic model of MS patients.

## Results

### Identification of differentially expressed gene between MS samples and healthy controls samples

**Fig. 1:**
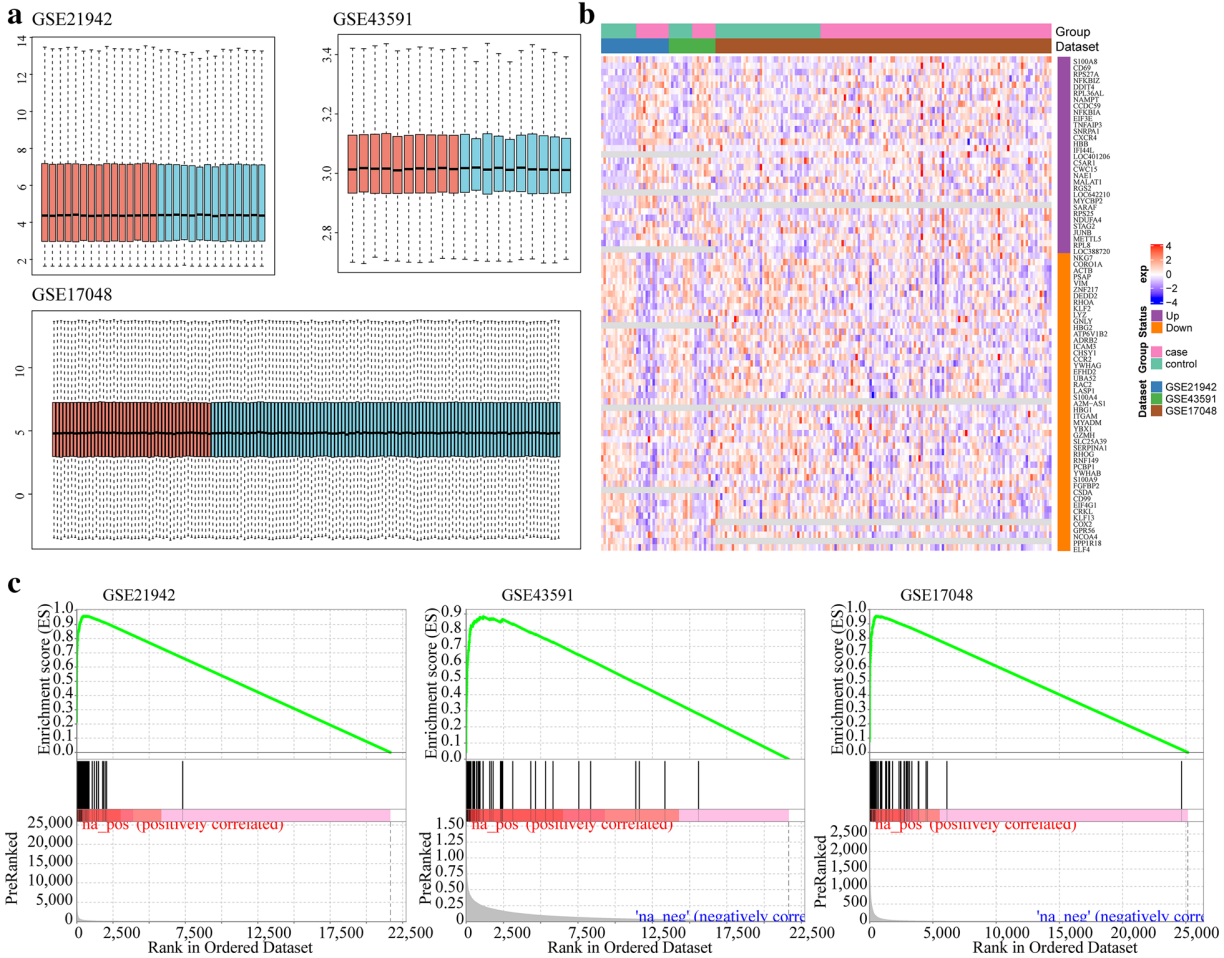
**a** Boxplots of overall expression levels of sample after standardization (blue: normal samples, red: MS samples). **b** Heatmap of DEGs in integrated microarray analysis, where the color in each rectangle represents the value of log2(foldchange), each row represents a dataset, and each column represents a gene. The gradient color from blue to red represents the change from down- to up-regulation. **c** Enrichment results for DEGs in the three datasets

Data sets GSE21942, GSE43591 and GSE17048 were obtained from GEO and standardized for consistent data distribution (Fig. 1a). 78 differentially expressed gene (DEGs) were finally screened after data pre-processing and quality control. Among the 78 DEGs, 47 genes were down-regulated in the disease group, while 31 genes were down-regulated in the healthy group, and the fold change heatmap of these DEGs in each dataset is shown in Fig. 1b. The GSEA enrichment results of the DEGs in each data set are shown in Fig. 1c, and it can be seen that DEGs were mainly enriched in the group with high fold change in each data set.

### Functional enrichment analysis of DEGs

**Fig. 2:**
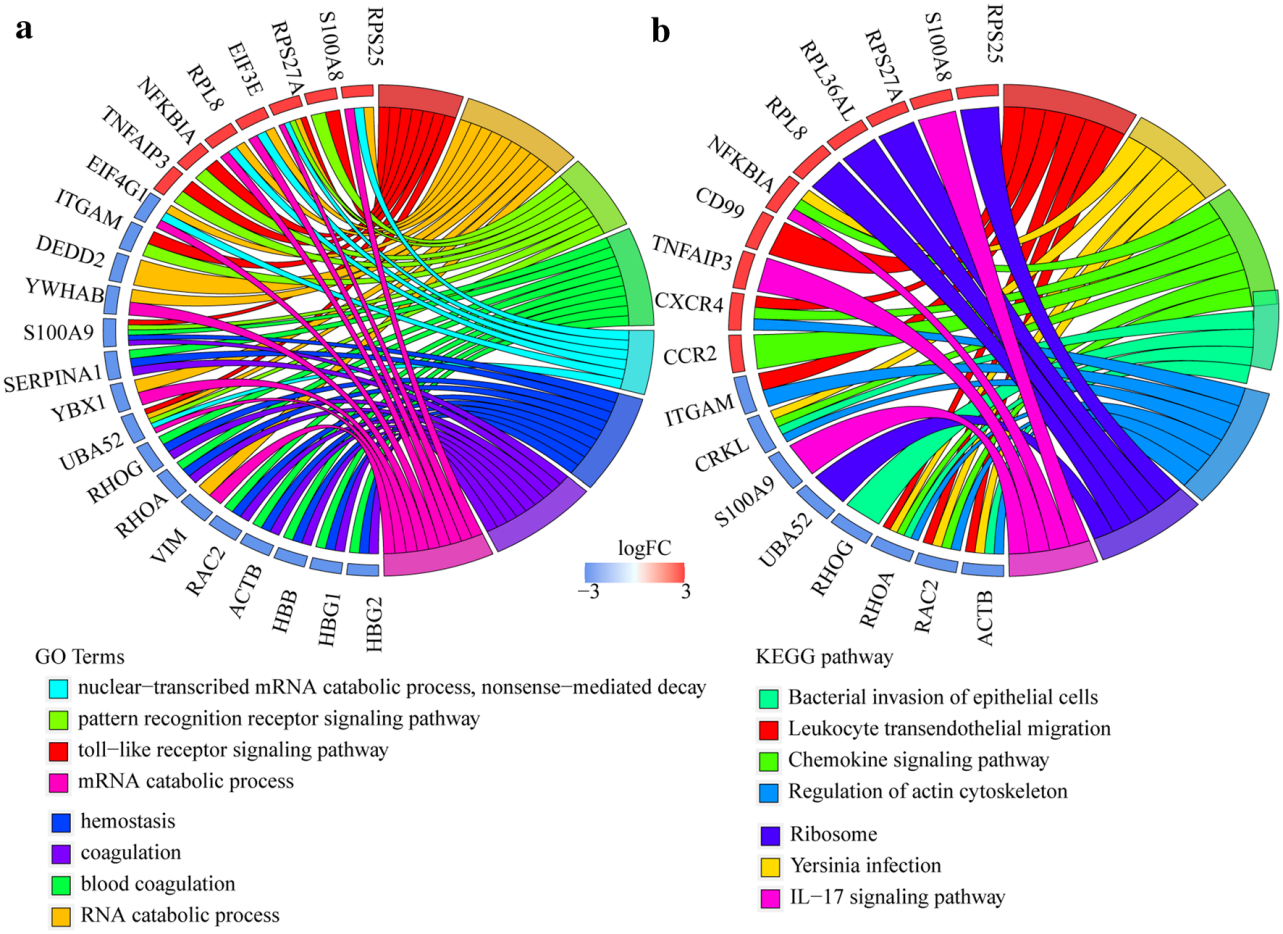
Functional enrichment analysis of 78 DEGs. **a** Enriched GO terms of DEGs. **b** Enriched KEGG biological pathways of DEGs. Different colors represent different functions, and the hyphen represents gene correspondence enrichment to GO term or KEGG biological pathways

To better understand the functional involvement of the DEGs, GO and KEGG functional enrichment analysis were performed on the 78 DEGs. The results showed 186 enriched GO terms, which were mainly enriched to toll-like receptor signaling pathway, pattern recognition receptor signaling pathway, innate immune response-activating signal transduction (Fig. 2a). Interestingly, these enriched pathways are important biological pathways involved in the immune process, and this is consistent with the nature of MS as an immune system disease. In addition, these genes were also enriched to bacterial invasion of epithelial cells, leukocyte transendothelial migration, chemokine signaling pathway, regulation of actin cytoskeleton, yersinia infection, ribosome, and IL-17 signaling pathway (Fig. 2b). Several of above listed pathways are associated with the development of MS, such as the IL-17 signaling pathway, which plays an important role in the pathogenesis of many autoimmune diseases including MS [24], and leukocyte transendothelial migration, which is a key feature of MS pathology [25].

### Identification of potential biomarkers associated with MS diagnosis

**Fig. 3:**
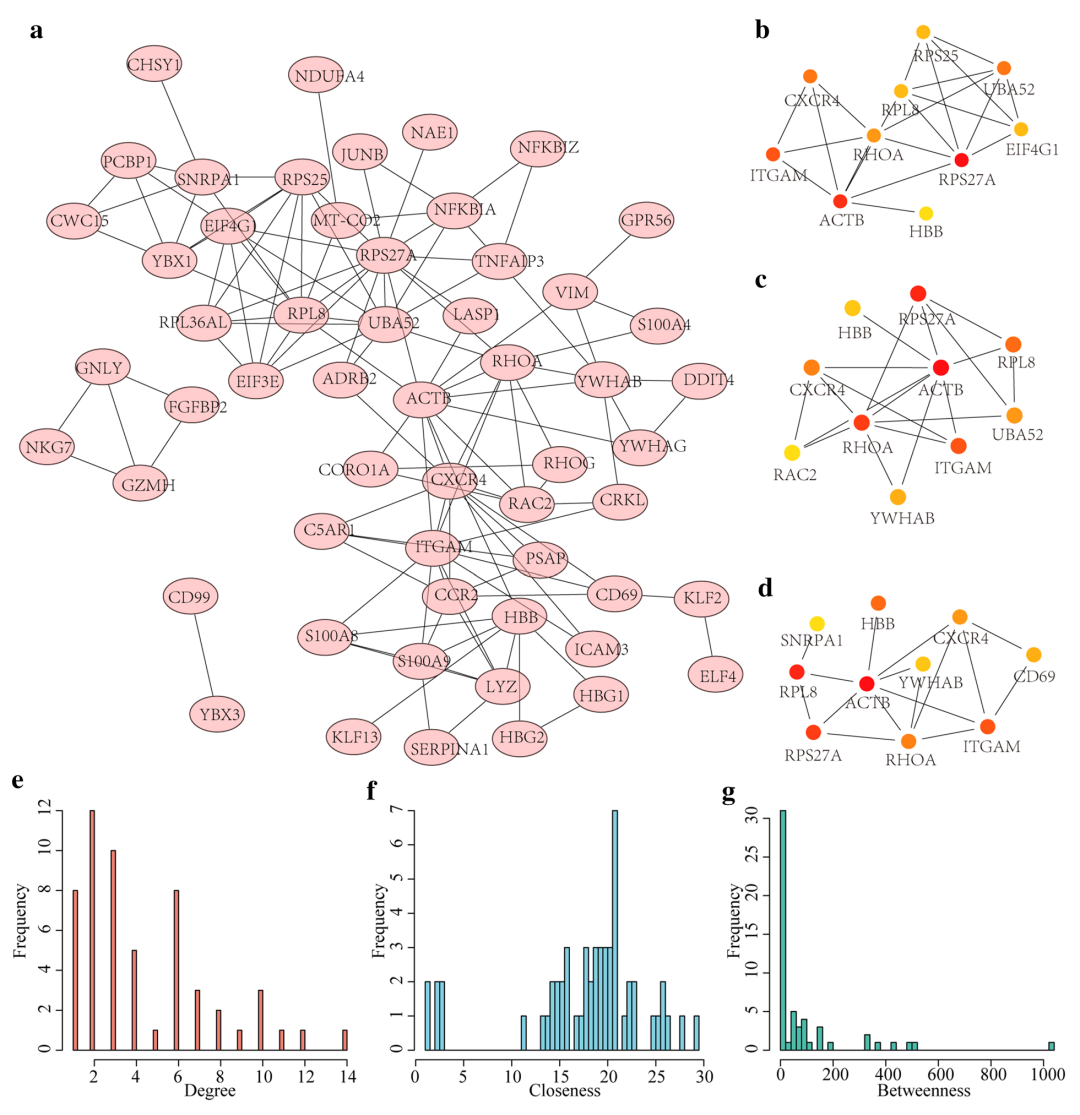
PPI protein interaction network analysis. **a** The protein interaction network of DEGs. **b** Hub nodes identified by the Degree method. **c** Hub nodes identified by the Closeness method. **d** Hub nodes identified by the Betweenness method. **e** Degree distribution of the network. **f** Closeness distribution of the network. **g** Betweenness distribution of the network. In **b–d**, the redder of the node in the network, the higher of the score

To examine the interactions among the 78 DEGs, each gene was mapped into the String database to obtain gene interactions, according to the threshold of a minimum required interaction score > 0.4 (medium confidence). After visualizing the gene interactions by Cytoscope, we found that the 78 DEGs were mapped into a total of 124 interactions in the network (Fig. 3a). The cytoHubba plugin in Cytoscope was used for hub gene identification, with the employment of three calculation methods (Degree, Closeness and Betweenness). The sub-networks of the top 10 genes evaluated by the three calculation methods are shown in Fig. 3b–d. It can be seen that hub genes with high degree, closeness and betweenness were generally consistent and showed interaction with multiple genes in the network. We further analyzed the topological properties of the network, and found that the distribution of degrees in the network (Fig. 3e) demonstrated a power-law distribution, which is consistent with biological network characteristics, as most of the gene degrees were less than 7. In addition, by calculating the closeness of the network, we found that most of the nodes have an overall high closeness of basically above 15 (Fig. 3f). Finally, the betweenness of the network was calculated and most nodes had a betweenness below 100 (Fig. 3g). Nodes with high degree, closeness or betweenness were considered as important nodes in the network. Top 5%,10%,15%,20%,25%,30% of the nodes with the highest degree, Closeness, and Betweenness as hub genes of the network were selected respectively, and GSE17048 was used as the training set to observe the classification performance under different thresholds (Additional file 1: Figure S1A). With the increase of the threshold, the number of included genes gradually increased, and the AUC also gradually increased. When the AUC rose slowly after 20%, we chose the top 20% as the threshold. By selecting the top 20% of the nodes with the highest degree, closeness, and betweenness as hub genes of the network, here, seven hub genes, namely, CXCR4, ITGAM, ACTB, RHOA, RPS27A, UBA52, and RPL8, were determined. Specifically, CXCR4 has multifunctional effects, and is widely involved in a variety of pathological conditions, including immune diseases, viral infections and cancer [26]. RhoA, which is a ubiquitously expressed cytoplasmic protein, belongs to the small GTPase family of enzymes, and acts as a molecular switch and is activated in response to the binding of chemokines, cytokines and growth factors. Mutations in Rho and Rho regulatory factors predispose to autoimmune diseases and are the cause of malignancies of the hematopoietic system [27]. These findings suggest that the seven hub genes could serve as potential biomarkers for MS, and that it is effective to mine MS-associated markers by constructing MS-specific protein interaction networks.

### Construction and verification of the MS diagnostic model

**Fig. 4:**
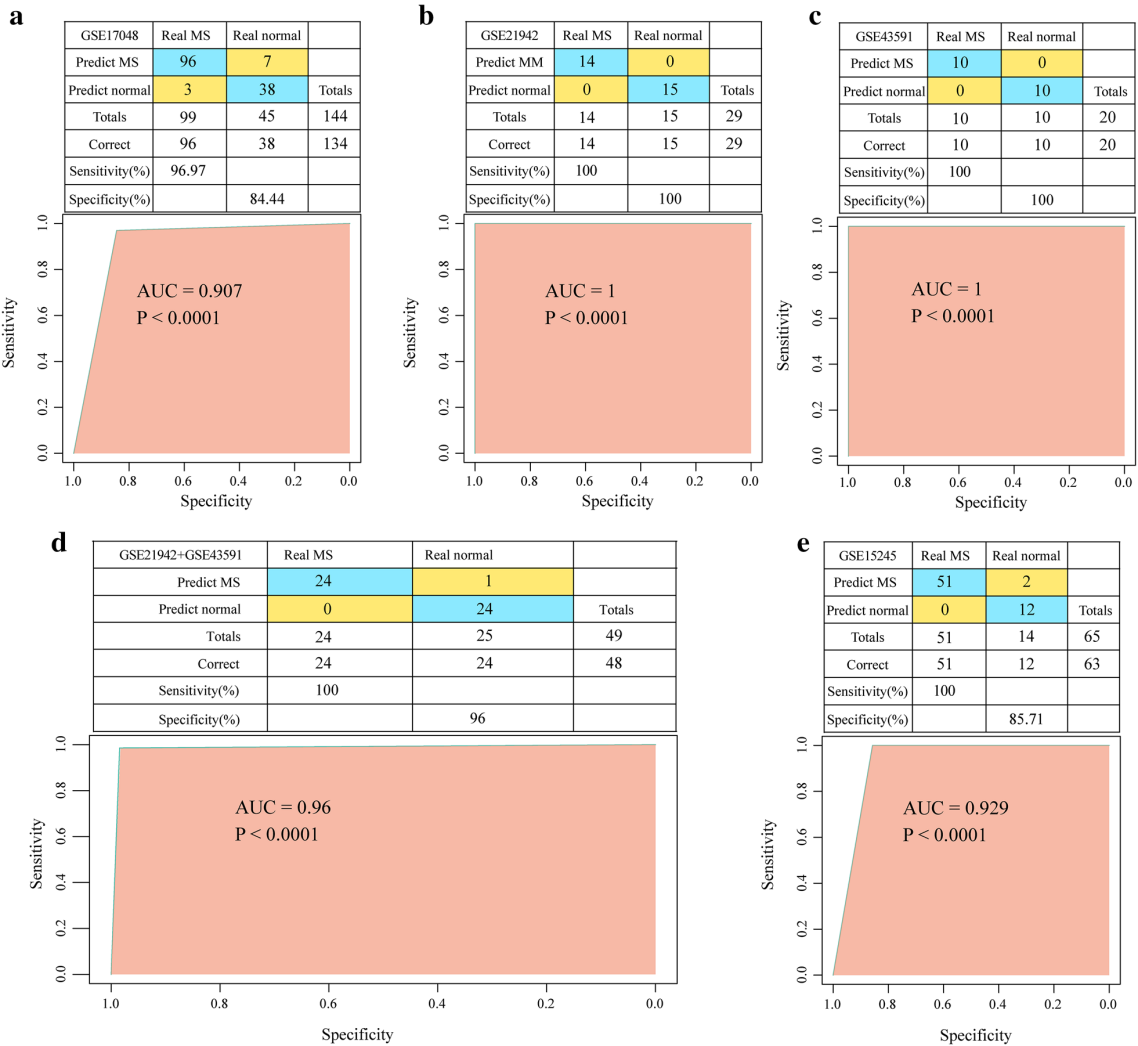
Construction of diagnostic model and validation of model. **a** Classification results and ROC curves of samples by diagnostic model in training data set. **b** Classification results and ROC curves of samples by diagnostic model in GSE21942. **c** Classification results and ROC curves of samples by diagnostic model in GSE43591. **d** Classification results and ROC curves of samples by diagnostic model in GSE43591 + GSE21942. **e** Classification results and ROC curves of samples by diagnostic model in GSE15245

**Fig. 5:**
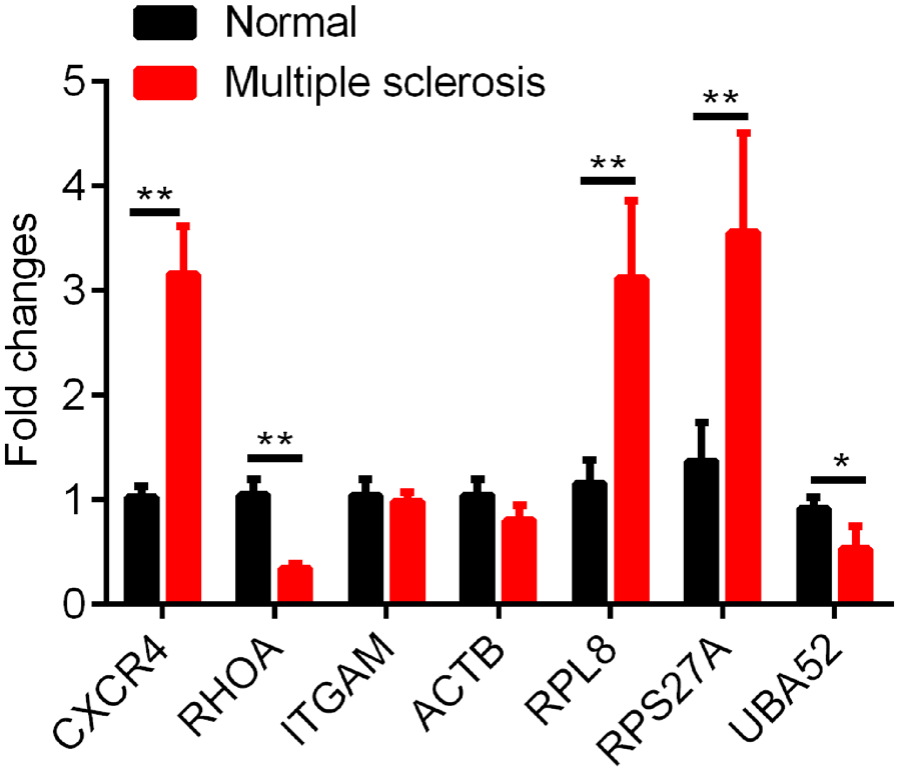
Differential expression analysis of CXCR4, ITGAM, ACTB, RHOA RPS27A, UBA52, and RPL8 in MS patient PBMC using RT-qPCR assay

We used GSE17048 as a training dataset (N = 144, MS = 99, Normal = 45), GSE21942 (N = 29, MS = 14, Normal = 15) and GSE43591 (N = 20, MS = 10, Normal = 10) as validation sets, and GSE15245 (N = 65, MS = 51, Normal = 14) as the independent validation set. Seven hub genes served as features in training data set, and their corresponding gene expression profiles were obtained. Then, the classification model was established by support vector machine (SVM). By applying tenfold cross-validation in the model test, 134 out of the 144 samples were correctly classified, with a classification accuracy of 93.06%, model sensitivity to MS of 96.97%, specificity of 84.44%, and area under the ROC curve (AUC) was 0.907 (Fig. 4a). Furthermore, the established model was used to predict the samples in the validation data set to test the prediction ability of the model. In the validation datasets GSE21942 and GSE43591, all the samples were correctly classified, with a classification accuracy of 100%, moreover, the sensitivity and specificity of the model for MS were all 100%, and the area under the receiver operating characteristic (ROC) curve was 1 (Fig. 4b, c). We merged the GSE21942 and GSE43591 datasets and applied the model to the merged dataset with an AUC of 0.96 (Fig. 4d). In the independent validation set GSE15245, 63 out of 65 samples were correctly classified with 96.92% classification accuracy, and the sensitivity and specificity of the model for MS were 100% and 85.71%, respectively, and the area under the ROC curve (AUC) was 0.929 (Fig. 4e). In addition, in the GSE15245 data set, the model was applied to samples of different genders, and it was observed that the AUC of Male samples was 0.916 (Additional file 2: Figure S2A) and that of Female samples was 0.91 (Additional file 2: Figure S2B), indicating that the prediction performance was similar in samples of different genders. According to the age distribution of the sample, the AUC of the model in the > 30 years sample was 0.9 (Additional file 2: Figure S2C) and the AUC of the model in the <  = 30 years sample was 0.88 (Additional file 2: Figure S2D), suggesting that the predictive performance of the model was similar across age groups. To further confirm the role of the seven genes, RT-qPCR was performed to detect the expressions of genes in the PBMC samples of MS patients. We found that in the PBMC samples of MS patients when compared with normal samples, CXCR4, RPL8 and RPS27A were obviously up-regulated, and UBA52 and RHOA were down-regulated (Fig. 5). Though the sample size may be relatively small, these results indicated that the diagnostic prediction model constructed in this study can effectively distinguish patients with MS from normal controls, and that the seven hub genes can be used as reliable biomarkers for MS diagnosis.

## Discussion

As is a chronic and progressive autoimmune disease, multiple sclerosis (MS) is a leading cause of disability to young adults [20]. However, the pathogenesis and progression of MS remain unclear. In this study, gene expression profiles of peripheral blood samples were analyzed by bioinformatics based on multiple microarray datasets. The results of functional enrichment analysis showed that differentially expressed genes were associated with Toll-like receptor signaling pathway, pattern recognition receptor signaling pathway, innate immune response-activating signal transduction, leukocyte transendothelial migration, IL-17 signaling pathway and immune-related signaling pathways. In MS, abnormalities of immune systems involve leukocyte transendothelial migration, IL-17 signaling pathway, and abnormalities of innate immune response-activating signal transduction pathway, here, innate and adaptive immunity play an important role [10, 15]. Moreover, immune dysregulation has been confirmed as an important mechanism in MS. It has been shown that leukocyte transendothelial migration is a driving factor in initiating an inflammatory immune response [28]. Above findings indicate that the pathogenesis of MS is multifactorial, complex, and is influenced by inflammation and external environment. CXCR4, ITGAM, ACTB, RHOA, RPS27A, UBA52, and RPL8 genes were identified as hub genes of the PPI network. Among them, CXCR4, ITGAM, ACTB, and RHOA are jointly involved in the Rap1 signaling pathway, leukocyte transendothelial migration, regulation of actin cytoskeleton processes; RPS27A, UBA52 and RPL8 genes are implicated in the ribosomal pathway (Additional file 1: Figure S1B).

CXCR4 is expressed in a variety of tissues, including in lymph nodes, brain, liver, colon, kidney, testis, lung, pancreas, skin and placenta, and in different cell types such as stromal cells, osteoblasts, fibroblasts, dendritic cells and monocytes [29, 30]. Focal areas of myelin destruction observed in MS often occur on a background of inflammation dominated by T-lymphocytes, hematogenous macrophages, microglial activation, and the presence of few B-lymphocytes and plasma cells [31, 32]. In vitro studies have shown that microglial activation leads to up-regulation of CXCR4 [33, 34].

ITGAM is a major non-human leukocyte antigen, and plays an important role in leukocyte activation, adhesion and migration through stimulated endothelium and in phagocytosis of complement-encapsulated granules and neutrophil apoptosis [35]. ITGAM is associated with the pathogenesis of systemic lupus erythematosus (SLE) [36], and an increasing number of studies have shown a genetic association between ITGAM and various autoimmune diseases [37–39]. RHOA is a ubiquitously expressed cytoplasmic protein that belongs to the small GTPase family. RhoA acts as a molecular switch and is activated in response to the binding of chemokines, cytokines and growth factors, moreover, as RhoA regulates the activation of cytoskeletal proteins and other factors through the mDia and ROCK signaling cascades, it is therefore regarded as a key regulator of innate and adaptive immunity [27]. In animal models of MS, absence of RhoA in T cells will reduce the number of mature T cells in the thymus and spleen, thereby significantly attenuating the incidence and severity of MS. RhoA is a central regulator of several prototypical T cell responses and a new potential therapeutic target for diseases such as MS [40]. ITGAM and RHOA showed a high degree and closeness in our PPI, therefore, we speculated that ITGAM and RHOA may be involved in MS through the regulation of T-cell activation.

ACTB encodes β-actin, an abundant cytoskeletal housekeeping protein. Sharp reduction of ACTB protein will change cell shape, migration, proliferation and gene expression, thereby impairing the development of the brain, heart and kidneys [41]. Pathogenic variants of ACTB are commonly associated with Baraitser-Winter prefrontal brain syndrome, resulting in severe, persistent dystonia, developmental delays and sensorineural hearing loss [42]. ACTB has long been considered an endogenous housekeeping gene and has been widely used as a reference gene/protein to reflect gene expression in tumors. Evidence increasingly demonstrated that ACTB is dysregulated in liver, melanoma, kidney, colorectal, gastric, pancreatic, esophageal, lung, breast, prostate, ovarian, leukemia and lymphoma. The aberrant expression and aggregation of ACTB and alterations in the cytoskeleton are associated with cancer aggressiveness and metastasis [43], suggesting that ACTB may be expressed at different levels in different environments and under different experimental conditions.

So far, RPS27A, UBA52, and RPL8 have not been reported in MS, and our study suggests that these genes are worthy of further study in MS.

Support vector machine (SVM) is a popular machine learning method widely used with biomedical data analysis. In this study, a diagnostic model of MS was developed based on SVM using the expression profiles of seven potential markers, and had an AUC of 0.907 in the training set, suggesting the accuracy of using these seven hub genes in classifying MS. We first validated the model in the dataset GSE21942 of the same platform with an AUC of 1 and an accuracy of 100%, and further validated the model in the dataset GSE43591 of a different platform and obtained an AUC of 1. Such results indicated the applicability of our diagnostic model to data from different chip platforms. Finally, to examine the model's prediction of data from different sources, the dataset GSE15245 originated from blood samples was applied for verification and an AUC of 0.929 was obtained, which indicates the reproducibility of the model. Moreover, the 7-gene diagnostic model can also be used to classify patients based on blood samples, thus, our model may have broad application prospects in clinical practice.

Although we used bioinformatics techniques to identify potential candidate markers involved in MS occurrence from a large sample, the study also has several limitations. Firstly, the sample lacked clinical follow-up information, thus, we did not consider factors such as the presence of other health states of the patient in affecting the identification of diagnostic biomarkers from the samples. Secondly, the results obtained only by bioinformatics analysis will require further experimental validation. Third, the study does not include any clinically isolated syndrome (CIS) cases or patients with other neurological diseases that make up the differential diagnoses for MS. Lastly, the model only benefited for MS patients. Therefore, further genetic and experimental studies with larger sample sizes and experimental validation are needed.

## Conclusions

In summary, in this study, we systematically analyzed the gene expression profiles of 248 blood samples, and constructed an aberrantly expressed gene signature involving a variety of important biological pathways for MS. Finally, we determined seven potential markers for MS based on protein interaction networks and developed a highly accurate diagnostic model, which is applicable to different microarray platforms and can be used in blood samples (mean AUC = 0.96). The findings of this study provide targets and references for clinicians and bio-laboratory scientists.

## Methods

**Fig. 6:**
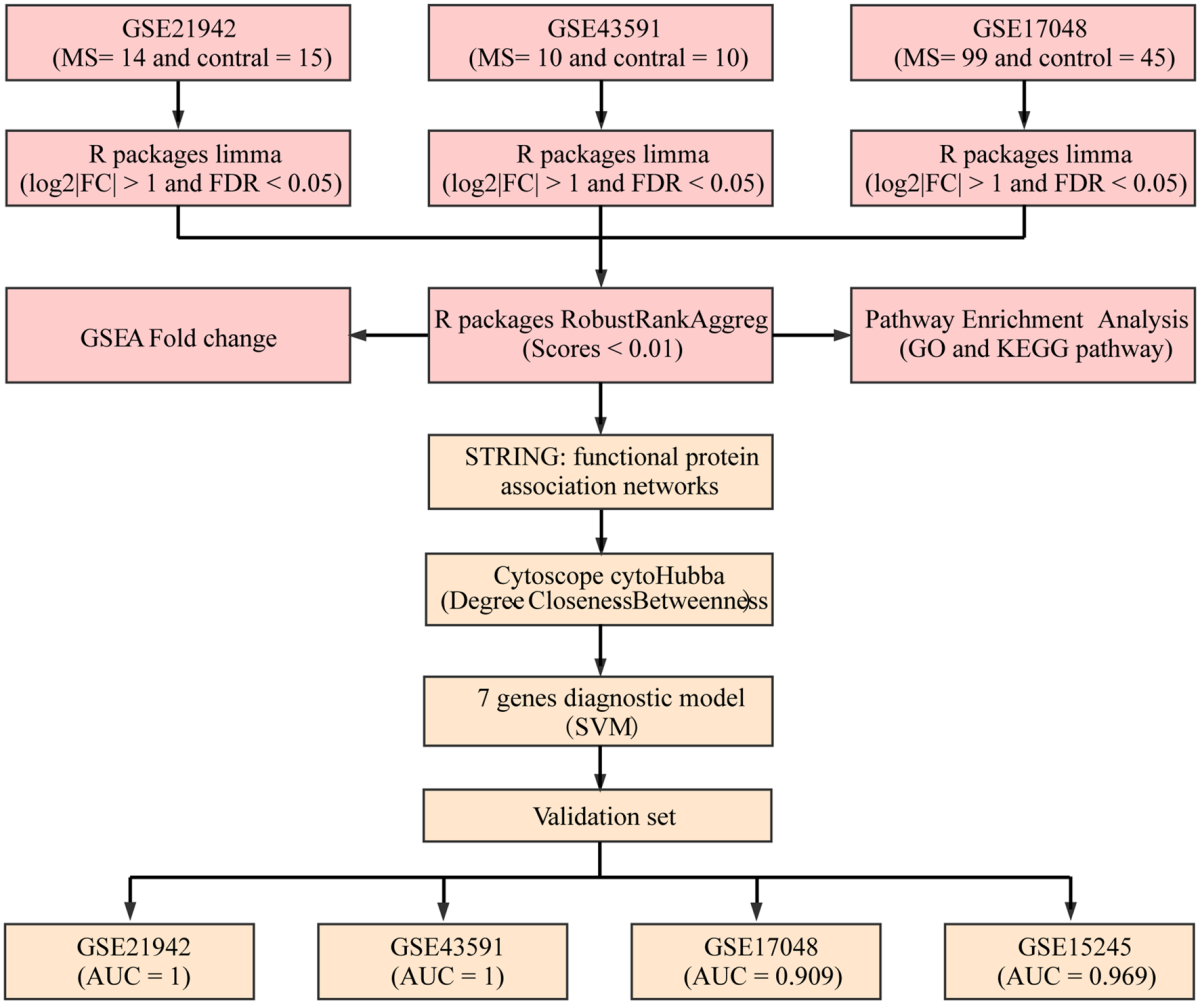
The workflow of the study

Briefly, this study began with data collection and differential expression analysis, and multiple data integration analysis to identify key differential genes, followed by function enrichment analysis, protein interaction network construction, feature selection, and construction and validation of the classifier (Fig. 6).

### Data collection

The NCBI GEO database was systematically searched to identify GEO datasets with microarray expression data relevant to MS. Only studies conducted with peripheral blood were included in our analysis. Data sets with < 10 samples and studies without control samples were excluded. MS pathology types were PPMS/RRMS/SPMS/CIS and samples derived from patients prior to drug treatment were included to subsequent analyses. Finally, four datasets (GSE21942, GSE43591, GSE15245, and GSE17048) were screened. GSE21942 and GSE43591 were downloaded from Affymetrix Human Genome U133 Plus 2.0 Array platform, GSE15245 and GSE17048 were respectively downloaded from Affymetrix Human Genome U133A 2.0 Array platform data and Illumina HumanHT-12 V3.0 expression beadchip. We obtained standardized expression profiling data of 84 control samples and 174 MS samples from the GEO database, and the sample information for each dataset was shown in Table 1. The GSE21942, GSE43591, and GSE15245 data sets are standardized by RMA, and the GSE17048 data sets are standardized by cubic spline. The probes were matched to genes, and those matched to multiple genes were removed. When multiple probes matched to one gene, the median of these probes were taken as the expression value for the gene. Here, we obtained four gene expression profiles.

**Table 1:** Studies and data included in this analysis

GEO accession	Sample size		Sample source	Platform
	MS case	Control		
GSE21942	14	15	Peripheral blood	GPL570 Affymetrix Human Genome U133 Plus 2.0 Array
GSE43591	10	10	Peripheral blood	GPL570 Affymetrix Human Genome U133 Plus 2.0 Array
GSE15245	51	14	Peripheral blood	GPL571 Affymetrix Human Genome U133A 2.0 Array
GSE17048	99	45	Peripheral blood	GPL6947 Illumina HumanHT-12 V3.0 expression beadchip
Total	174	84		

### Integration of multiple data sets to identify differentially expressed genes

The differentially expressed genes (DEGs) between normal samples and MS samples were screened by R software package limma [44] in GSE21942, GSE43591 and GSE17048. To include more genes with high differences, FDR < 0.05 and |log2(Foldchange)|> 1 was used as the thresholds to screen DEGs. The R package RobustRankAggreg [45] was used to integrate the DEGs screened from the three gene expression profiles, and genes with a score value less than 0.01 were considered as the DEGs. The distribution of fold change of DEGs in different data sets and the enrichment relationship of DEGs in different data sets were examined by performing GSEA analysis of DEGs using the rank order of fold change of genes in each data set as a background.

### Functional enrichment analyses

Gene Ontology (GO) and Kyoto Encyclopedia of Genes and Genomes (KEGG) pathway enrichment analysis were performed using the clusterProfiler [46] for analyzing the gene relationship with DEGs. Subsequently, over-represented GO terms in biological processes and KEGG pathway were identified and visualized using the R package GOplot [47]. For both analyses, FDR < 0.05 was considered to denote statistical significance.

### Protein interaction network construction and identification of key genes

The STRING Database (https://string-db.org/), which is an online platform for predicting gene interactions, is designed to collect, evaluate and integrate all public "protein–protein" interaction resources and to complement the results of computer predictions [48]. To analyze the interaction correlations of DEGs, we mapped DEGs to the STRING (version 11.0) database to obtain the interaction relationships among the genes. A combined score > 0.4 served as the threshold to establish a PPI network, and then the topological properties of the network was visualized and analyzed by Cytoscape24 software (version 3.7.1) [49]. In addition, the plug-in cytoHubba25 [50] in Cytoscape software was used to calculate the network's degree, closeness and betweenness for identifying key genes from the PPI network.

### Construction of MS diagnostic prediction model and assessment of predictive performance of the model

Support Vector Machine (SVM) is a supervised learning model for machine learning algorithms and can be used to analyze data and identify gene expression patterns. The SVM could construct a hyperplane in high or infinite dimensional space for classification and regression. Specifically, based on a set of training samples with each marker belonging to one of two categories, a SVM training algorithm builds a model that assigns new instances to one or multiple categories, making it a non-probabilistic binary linear classification. Here, according to the expression profiles of key genes, we constructed a diagnostic prediction model based on SVM classification (51). We used GSE17048 as a training dataset, and GSE21942 and GSE43591 as validation sets. Additionally, GSE15245 served as an independent set of external validation sets to validate the prediction performance of the model. The model was constructed in the training dataset and its classification capability was validated using a tenfold cross-validation method. Samples from the validation dataset were predicted using the built model. The predictive ability of the model was reflected by the area under the ROC curve (AUC), and the sensitivity and specificity of the model to predict MS patients were analyzed.

### RT-qPCR

TRIzol (Thermo, 15,596,026) were applied to extract total RNA from peripheral blood mononuclear cell (PBMC), included 4 MS patients and 5 control samples. The synthesis of total RNA into cDNA was conducted according to the instructions of the reverse transcription kit (Thermo, BTK1622). Amplification was performed by conducting real-time polymerase chain reaction in an ABI 7500 real-time PCR instrument with an one-step qRT-PCR Kit (FP303, Tiangen Biochemical Technology (Beijing) Co., Ltd.). The primers were as follows: CXCR4 forward, CTTGACACTGGATATACACTTCAG and reverse, AACAGGGTTCCTTCATGGAG; ITGAM forward, CAATATCAGGTCAGCAACCTG and reverse, ATGACAGTCTGGTTCAGCC; ACTB forward, GAAGATCAAGATCATTGCTCCTC and reverse, ATCCACATCTGCTGGAAGG; RHOA forward, AGTTCCCAGAGGTGTATGTG and reverse, CCAACTCTACCTGCTTTCCA; GAPDH forward, TCAAGATCATCAGCAATGCC and reverse, CGATACCAAAGTTGTCATGGA; GAPDH was an internal control. 2^−△△Ct^ was used to calculate the relative expressions. The T test was used to analyze the differences in gene expression between the two groups.

## Supplementary Information

**Additional file 1: Figure S1.** ROC analysis and KEGG analysis. **A** AUC of gene combinations identified under different thresholds in the training set. **B** The KEGG Pathway where CXCR4, ITGAM, ACTB, RHOA, RPS27A, UBA52 and RPL8 genes participate together.

**Additional file 2: Figure S2.** Classification performance of MS diagnostic model in genders and ages in GSE15245 dataset. **A** The classification result and ROC curve of the MS sample of the diagnostic model in the Male sample; **B** The classification result and ROC curve of the MS sample of the diagnostic model in the Female sample; **C** The classification result and ROC curve of the MS sample of the diagnostic model in the Age > 30 samples; **D** The classification result and ROC curve of the MS sample of the diagnostic model in the v samples.

## Data Availability

The datasets used and/or analyzed during the current study are available from the corresponding author on reasonable request.

## Abbreviations

MS: Multiple sclerosis
PPI: Protein–protein interaction
SVM: Support vector machine approach
EBV: Epstein–Barr virus
CNS: Central nervous system
BBB: Blood–brain barrier
DEGs: Differentially expressed genes
GO: Gene Ontology
KEGG: Kyoto Encyclopedia of Genes and Genomes
