# Supplementary figures and images for: Identification of blood-derived candidate gene markers and a new 7-gene diagnostic model for multiple sclerosis
Source: Biol Res. 2021 Apr 1;54:12. doi: 10.1186/s40659-021-00334-6 (PMC8015180; doi:10.1186/s40659-021-00334-6)

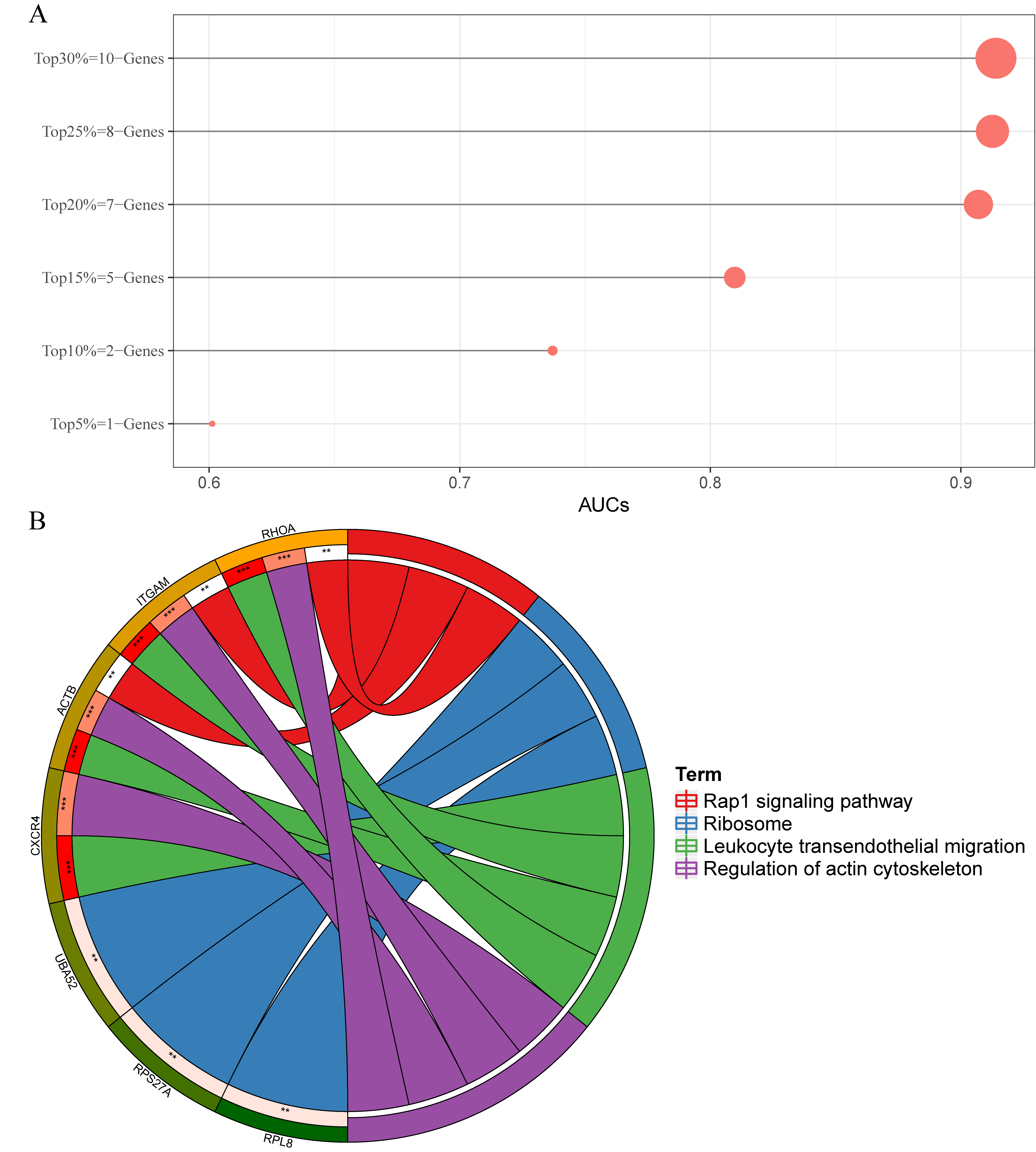

Supplement: Supplementary file 1 — Additional file 1: Figure S1. ROC analysis and KEGG analysis. A AUC of gene combinations identified under different thresholds in the training set. B The KEGG Pathway where CXCR4, ITGAM, ACTB, RHOA, RPS27A, UBA52 and RPL8 genes participate together. [file 40659_2021_334_MOESM1_ESM.tif]

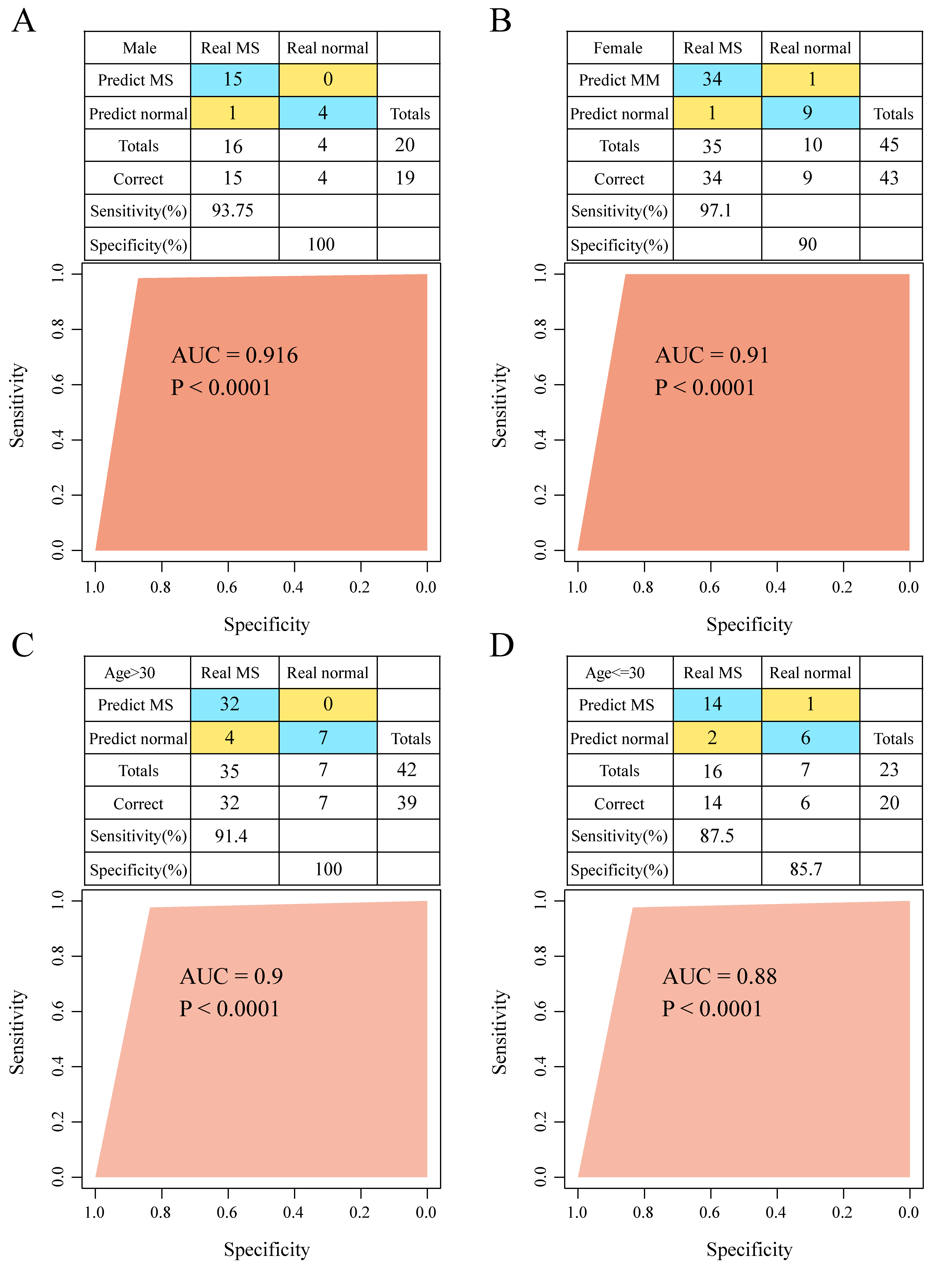

Supplement: Supplementary file 2 — Additional file 2: Figure S2. Classification performance of MS diagnostic model in genders and ages in GSE15245 dataset. A The classification result and ROC curve of the MS sample of the diagnostic model in the Male sample; B The classification result and ROC curve of the MS sample of the diagnostic model in the Female sample; C The classification result and ROC curve of the MS sample of the diagnostic model in the Age > 30 samples; D The classification result and ROC curve of the MS sample of the diagnostic model in the v samples. [file 40659_2021_334_MOESM2_ESM.tif]
